# 3D-printed Sr_2_ZnSi_2_O_7_ scaffold facilitates vascularized bone regeneration through macrophage immunomodulation

**DOI:** 10.3389/fbioe.2022.1007535

**Published:** 2022-09-16

**Authors:** Hao Pan, Li Deng, Lingwei Huang, Qi Zhang, Jing Yu, Yueyue Huang, Lei Chen, Jiang Chang

**Affiliations:** ^1^ Joint Centre of Translational Medicine, the First Affiliated Hospital of Wenzhou Medical University, Wenzhou, Zhejiang, China; ^2^ Department of Orthopaedics, The First Affiliated Hospital of Wenzhou Medical University, Wenzhou, Zhejiang, China; ^3^ Wenzhou Institute, University of CAS, Wenzhou, Zhejiang, China; ^4^ Oujiang Laboratory, Wenzhou, Zhejiang, China; ^5^ Key Laboratory of Intelligent Treatment and Life Support for Critical Diseases of Zhejiang Province, Wenzhou, Zhejiang, China

**Keywords:** 3D printing, immunomodulation, osteogenesis, angiogenesis, bone defect

## Abstract

Biomaterial-based bone grafts are emerged as an effective strategy for the treatment of large bone defects, especially for the scaffolds with enhanced osteogenic and angiogenic bioactivities. However, most studies focused on the direct interactions between scaffolds and bone-related cells such as osteoblasts and endothelial cells, and ignored the effects of material-triggered immunomodulation and the subsequent immune-regulated bone regeneration process. In this study, we developed a silicate bioceramic (Sr_2_ZnSi_2_O_7_, SZS) scaffold with well-defined pore structures using a three-dimensional (3D) printing technique. The prepared scaffolds were biodegradable, and the released bioactive ions were beneficial for immunomodulation, which stimulated macrophages to release more pro-healing cytokines and less pro-inflammatory cytokines. The obtained scaffold/macrophage conditioned medium further promoted the proliferation and osteogenic differentiation of a murine preosteoblast cell line (MC3T3-E1), as well as the angiogenic activity of human umbilical vein endothelial cells (HUVECs). Moreover, the *in vivo* experiments of critical-sized calvarial defects in rats revealed that the 3D printed SZS scaffolds could facilitate more vascularized bone regeneration than the 3D printed β-tricalcium phosphate (β-TCP, a typical clinically used bioceramic) scaffolds, suggesting that the 3D-printed SZS scaffolds hold the potential as implantable biomaterials with favorable osteoimmunomodulation for bone repair.

## 1 Introduction

Large bone defect is a common but serious medical problem in clinical practice, which is mostly caused by trauma, infection, or tumor resection and can’t be self-healed when the lesion exceeds the “critical size” (Roddy et al., 2018; Sparks et al., 2020). Bone grafts are usually needed for the reconstruction of large bone defects, serving as an osteoconductive scaffold for new bone formation. The currently available bone grafts mainly include biological products (autologous or allogeneic implants), and synthetic biomaterials (Yang et al., 2018; Wei et al., 2022). Although both autologous and allogeneic bone grafts have been implemented clinically for many years and proved as effective strategies for bone restoration, their major disadvantages are still unsolved. For example, autologous bone grafting needs additional surgical intervention and is subject to the limited bone supply, while allogeneic bone grafting has the risk of immunological rejection and disease propagation (Zhao et al., 2021). Therefore, it is imperative to develop suitable artificial bone scaffolds for the repair of large bone defects.

An ideal bone scaffold requires many properties, among which the bioactivity of osteogenesis and angiogenesis are two of the principal elements (Yang et al., 2021b). Various methods have been developed to promote the osteogenic and angiogenic ability of the scaffold simultaneously (Shi et al., 2015; Feng et al., 2017; Kuttappan et al., 2018; Yang et al., 2019). Among these methods, modifying scaffolds with growth factors is the most direct way. For example, Yasuhiko Tabata *et al.* developed a fibrous scaffold loaded with both bone morphogenetic protein 2 (BMP-2, an osteogenic growth factor) and vascular endothelial growth factor (VEGF, an angiogenic growth factor) for the critical-sized calvarial defect in rats (Kuttappan et al., 2018). The results showed that the co-delivery of BMP-2 and VEGF by the scaffolds significantly stimulated the vascularized bone regeneration in the defect area. However, the drawbacks of high cost, instability and short life hinder the translational application of growth factors. Another commonly used strategy for enhancing osteogenesis and angiogenesis is based on material-mediated immune regulation. In general, bone reconstruction is a result of the interplay between the skeletal and immune systems, and the implanted scaffold-triggered inflammation has been recognized as the first stage of bone regeneration, which markedly affects the following bone healing and remodeling process (Chen et al., 2016). It has been widely proved that a beneficial immune microenvironment for osteogenesis and angiogenesis could be modulated by tailoring the composition or structure of a scaffold (Yang et al., 2019; Negrescu and Cimpean, 2021). For example, in our previous study, we proved that the decoration of micro/nano hierarchical structures on a hydroxyapatite (HA) ceramic could promote the polarization of macrophages from phenotype M1 towards M2, showing benefits for the pro-healing immune environment, which further increased the expressions of osteogenic genes in human bone marrow stromal cells (hBMSCs) and angiogenic genes in human umbilical vein endothelial cells (HUVECs) (Yang et al., 2019).

Apart from the structure of the implanted scaffolds, the component of the material is also a key factor in the regulation of the immune microenvironment. Scaffolds containing trace elements such as silicon (Si), zinc (Zn), and strontium (Sr) and has shown great potential in manipulating the immune microenvironment by affecting multiple immune cells including macrophages and Treg cells (Liu et al., 2018; Bai et al., 2021; Zhang et al., 2021; Zhong et al., 2022). In our previous study, a bioactive ceramic (Sr_2_ZnSi_2_O_7_, SZS) containing Si, Zn, and Sr was synthesized and plasma-sprayed on a titanium alloy implant, which significantly inhibited the release of pro-inflammatory cytokines, showing a beneficial osteoimmmunomodulation due to sustaining released of silicate ions (SiO_3_^2-^), zinc ions (Zn^2+^) and strontium ions (Sr^2+^) (Chen et al., 2014). However, it is unknown if the ions-regulated immune microenvironment is also beneficial for angiogenesis. Also, titanium alloy is non-biodegradable, which may require surgical removal due to possible complications such as infection and exposure. In contrast, a degradable SZS scaffold itself might be more suitable for bone regeneration. Therefore, in this study, we prepared SZS scaffolds by three-dimensional (3D) printing and investigated their effects on the immune microenvironment, as well as the following osteogenic and angiogenic potential both *in vitro* and *in vivo*. It is expected that the proposed SZS scaffolds could enhance osteogenesis and angiogenesis for bone tissue regeneration via immunomodulation.

## 2 Materials and methods

### 2.1 Preparation of SZS powders and scaffolds

SZS powders were compounded in a sol-gel method (Zhang et al., 2012). Briefly, nitric acid (HNO_3_, Aladdin Reagent Co., Ltd, Shanghai, China) was added to 400 ml distilled water to adjust pH to 2 followed by adding 0.4 mol TEOS and stirring for 1.5 h. Then, strontium nitrate (Sr(NO_3_)_2_, Aladdin Reagent Co., Ltd, Shanghai, China) and zinc nitrate hexahydrate (Zn(NO_3_)_2_·6H_2_O, Aladdin Reagent Co., Ltd, Shanghai, China) were added to the tetraethyl orthosilicate (TEOS, Aladdin Reagent Co., Ltd, Shanghai, China) solution in a molar Sr: Zn: Si ratio of 2:1:2 and stirred for 5 h. Sequentially, the mixture was put at 60°C for 24 h until gel formation, and the formed gel was stabilized at 60°C for another 1∼2 h, which was followed by drying the gel at 120°C, as well as milling and filtering to acquire particles less than 200-mesh. Finally, the obtained particles were sintered in a high temperature furnace (KSL-1700X-A4, HF-kejing Co., Ltd, Anhui, China) at 1,200°C for 3 h at a heating rate of 2°C/min and ground for further use.

For scaffolds, the printing paste was first prepared by mixing SZS powders (2 g) with sodium alginate (Alg, 0.01 g), and Pluronic®F-127 (F-127,2 ml, 20 wt%). Then, the 3D printer (BioMaker, SunP Biotech, Beijing, China) was applied to prepare porous SZS scaffolds with a 45°-crossed lay-down pattern at a printing speed of 4.4 mm/s. As a control, β-tricalcium phosphate (β-TCP) scaffolds were also 3D printed similarly. The obtained 3D printed ceramic embryos were further sintered at 1,200–1,400°C for SZS, and 1,100°C for β-TCP.

### 2.2 Characterization of the 3D printed scaffolds

The component of the SZS scaffolds was characterized using an X-ray diffractometer (XRD, Bruker, Germany) with 40 kV and 40 mA. The structure and surface morphology of the scaffolds were examined with a scanning electron microscope (SEM, SU8010, HITACHI, Japan). To study the release of SiO_3_^2-^, Zn^2+^ and Sr^2+^ ions from SZS scaffolds, SZS scaffolds were placed in a 48-well plate with 1 ml/well deionized water for 2 days, and the extracts were collected and filtered using Millipore filters with pore size of 0.22 µm before quantifying the concentration of these ions by an inductively coupled plasma-mass spectrometry (ICP-MS, Agilent7850, Singapore). The mechanical properties of the scaffolds were evaluated with a universal testing machine (Instron 5,944, America). The porosity (P) of the scaffolds was measured using a drainage method (Yang et al., 2017). Briefly, the weight of the scaffolds was measured in dried (M1), water-filled (M2) and water-immersion (M3) conditions, respectively. The porosity (P) of the scaffolds was calculated by the following formula.

$$P=\frac{M2-M1}{M2-M3}\times 100\%$$ (1)

For the degradation test, scaffolds were immersed into Tris-HCl buffer solution in a ratio of 50 ml/g at 37°C for 1, 3, and 7 days. Then, the scaffolds were weighed after drying at 120°C for 12 h. The degradation rate was calculated as the percentage of weight loss of scaffolds during the immersion.

### 2.3 Cell experiment

#### 2.3.1 Inflammatory reaction of the scaffolds

For the cytotoxicity assay, RAW264.7 macrophages were seeded on the scaffolds in a 48-well plate at a density of 1 × 10^4^ cells/well and cultured for 1 day. For, imflammatory reaction assay, RAW264.7 macrophages were seeded on scaffolds in a 6-well plate with a density of 2 × 10^5^ cells/well and cultured for 2 days. Then, the total RNA was extracted using an RNA kit (YISHEN, China), and transferred into cDNA using a reverse transcription kit (YISHEN, China). Gene expression of inflammation factors including interleukin-1α (IL-1α),interleukin-1β (IL-1β), inducible nitric oxide synthase (iNOS), tumor necrosis factor-α (TNF-α), transforming growth factor-1β (TGF-1β), interleukin-1 receptor antagonist (IL-1rα), cluster of differentiation 206 (CD206), arginine (ARG) was evaluated using a real-time polymerase chain reaction (RT-PCR) combined with SYBR Green PCR Master Mix (YISHEN, China). The gene expression was normalized to GADPH. The primer sequences are shown in Table 1.

**TABLE 1:** Primers for gene expression analysis.

Gene	Forward primer	Reverse primer
IL-1β	AAT​GCC​ACC​TTT​TGA​CAG​TGA​TG	TGA​TGT​GCT​GCT​GCG​AGA​TT
TNF-α	TAG​CCC​ACG​TCG​TAG​CAA​AC	GCA​GCC​TTG​TCC​CTT​GAA​GA
iNOS	ACC​CCT​TGT​GCT​GTT​CTC​AG	GGG​ATT​CTG​GAA​CAT​TCT​GTG​C
IL-1α	GTC​GGG​AGG​AGA​CGA​CTC​TAA	GTT​TCT​GGC​AAC​TCC​TTC​AGC
TGF-1β	TGA​TAC​GCC​TGA​GTG​GCT​GTC​T	CAC​AAG​AGC​AGT​GAG​CGC​TGA​A
IL-1rα	AGA​GCC​CCT​TAT​AGT​CAC​GAA	TAC​ACC​CTG​CAA​AAG​TTG​TTC​C
ARG	AAC​CTT​GGC​TTG​CTT​CGG​AAC​TC	GTT​CTG​TCT​GCT​TTG​CTG​TGA​TGC
CD206	ATC​CAC​GAG​CAA​ATG​TAC​CTC​A	TAG​CCA​GTT​CAG​ATA​CCG​GAA
RUNX-2	GAC​TGT​GGT​TAC​CGT​CAT​GGC	ACT​TGG​TTT​TTC​ATA​ACA​GCG​GA
COL-1	TTC​TCC​TGG​CAA​AGA​CGG​AC	CTC​AAG​GTC​ACG​GTC​ACG​AA
OCN	GAA​CAG​ACA​AGT​CCC​ACA​CAG​C	TCA​GCA​GAG​TGA​GCA​GAA​AGA​T
BMP-2	TCA​CTT​ATA​GCC​GCA​TTA​TCT​TCT​TC	TTG​GTT​TAT​CCA​TGA​GGC​TAA​CTG
bFGF	CAA​TTC​CCA​TGT​GCT​GTG​AC	ACC​TTG​ACC​TCT​CAG​CCT​CA
VEGF	TAT​GCG​GAT​CAA​ACC​TCA​CCA	CAC​AGG​GAT​TTT​TCT​TGT​CTT​GCT
FGF-2	AAA​AGG​CAA​GAT​GCA​GGA​GA	TTT​TGC​AGC​CTT​ACC​CAA​TC
HIF-1α	ATC​CAT​GTG​ACC​ATG​AGG​AAA​T	CTC​GGC​TAG​TTA​GGG​TAC​ACT​T
eNOS	GAT​GTT​ACC​ATG​GCA​ACC​AAC	GAA​AAT​GTC​TTC​GTG​GTA​GCG

#### 2.3.2 Effect of conditional medium on MC3T3-E1 cells

To prepare scaffold/macrophage conditional media, the supernatant from the cell experiment of 2.3.1 was collected and mixed with Dulbecco’s modified eagle medium (DMEM) at a ratio of 1:1. The following cell experiments were implemented under the stimulation of conditional media. For the cell proliferation study, MC3T3-E1 cells were seeded in a 96-well plate with a density of 1,000 cells/well, and cultured in humidified 5% CO_2_ at 37°C for 12 h. Then, the culture medium was replaced by the conditional medium, and the cells were cultured for 1, 3 and 5 days, during which the culture medium was exchanged every other day. Cell proliferation was evaluated by the cell counting kit-8 (CCK-8, YESEN, China) assay using a microplate reader (EPOCH2NS, BioTek instruments, America) at a wavelength of 450 nm. For the alkaline phosphatase (ALP) and alizarin red staining (ARS) experiments, cells were cultured with the conditional medium for 7 days and stained by kits purchased from Solarbio (Beijing, China). For the gene expression study, MC3T3-E1 cells were seeded in a 6-well plate with a density of 1 × 10^5^ cells/well and cultured in the conditional medium for 7 days. Then, the osteogenic genes of osteocalcin (OCN), type Ⅰ collagen (COL-1), runt-related transcription factor 2 (RUNX-2) and BMP-2 were evaluated following the same procedure above.

#### 2.3.3 Effect of conditional medium on HUVECs

The cell proliferation study of HUVECs under the conditional medium was assessed using a CCK-8 assay kit with an initial cell density of 1,000 cells/well in 96-well plates on day 1, 3 and 5. For the cell migration study, HUVECs were seeded in a 6-well plate with a density of 4 × 10^5^ cells/well and cultured for 12 h. Then, scratches were made with a pipette tip followed by phosphate buffered solution (PBS) washing twice. After microscopy, the conditional medium was added and cultured for another 24 h. The cells were imaged again after fixation with 4% paraformaldehyde and staining with methylrosanilnium chloride solution. The acquired images were processed using ImageJ software, where the original width (A_0_) and final width (A_1_) of the scratches were measured. The immigration rate (A) was calculated by the following formula.

$$A=\frac{A0-A1}{A0}\times 100\%$$ (2)

The *in vitro* tube formation experiment was conducted by seeding HUVECs (4 × 10^4^ cells/well) on matrigel (ABW®Matrigengel, China) in a 48-well plate and incubating with the conditional medium for 4 h. Microscopy images were taken using optical microscope (ckx53, Olympus, China) and analyzed with the ImageJ software (National Institutes of Health, United States ). In addition, the gene expression experiments of HUVECs were performed similarly to the above studies, and the angiogenic genes of vascular endothelial growth factor (VEGF), fibroblast growth fator 2 (FGF-2), basic fibroblast growth factor (bFGF), hypoxia inducible factor 1α (HIF-1α) and bmpendothelial nitric oxide synthase (eNOS) were evaluated.

### 2.4 Animal experiment

#### 2.4.1 Animal model and grouping

Sprague-Dawley mature male rats (8-week-old) were purchased from the Zhejiang Provincial Laboratory Animal Center, and the experiment was approved by the Animal Research and Ethics Committee of Wenzhou Institute of University of Chinese Academy of Sciences. A rat critical-sized cranial bone defect model was established according to a previous study (Yang et al., 2021c). Briefly, rats were anesthetized by intraperitoneal injection of pentobarbital. Then, full-thickness bone defects (5 mm) were created on both sides of the calvarium by a trephine drill. After implanting the 3D printed porous SZS (experimental group) or β-TCP (control group) scaffolds (with a diameter of 5 mm and a height of 1 mm) into the defects, the skin wound was sutured.

#### 2.4.2 Evaluation of bone regeneration

After an 8-weeks recovery, the rats were sacrificed and their skulls were collected for fixation with 4% paraformaldehyde and micro-computed tomography (micro-CT) scanning (Skyscan1176, Bruker, Germany). Bone mineral density and bone microstructure were quantified using the manufacturer’s analysis software (CTAn, Bruker, Germany). Then, the samples were decalcified with ethylenediamine tetraacetic acid (EDTA), dehydrated with gradient alcohol, embedded in paraffin, and sectioned into slices with a thickness of 4 μm. Histological analysis was performed following hematoxylin-eosin (H&E) staining and Masson’s trichrome staining, as well as immunohistochemistry staining of platelet endothelial cell adhesion molecule-1 (PECAM-1, CD31) and osteocalcin (OCN).

### 2.5 Statistical analysis

A one-way analysis of variance (ANOVA) was used to compare the difference between groups (≥3), and a Student’s t-test was used to analyze data between the two groups using GraphPad software. All the data were presented as mean ± SEM, and the significance level for tests was *p* < 0.05.

## 3 Results

### 3.1 Comparison of the SZS scaffolds sintered at different temperatures

**FIGURE 1:**
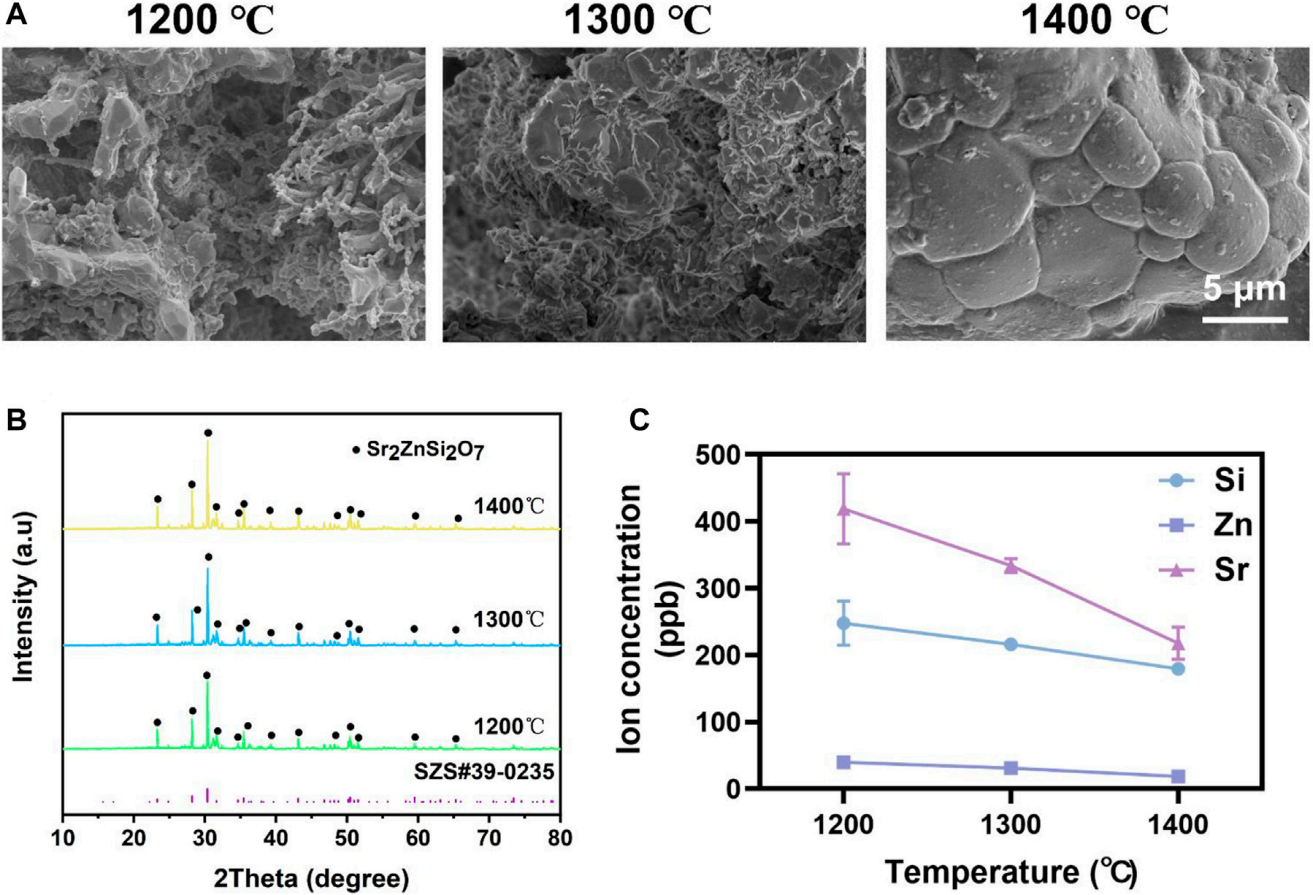
Characterization of SZS scaffolds sintered at 1,200°C, 1,300°C, and 1,400°C, respectively. **(A)** SEM characterization of the surface morphology. **(B)** XRD analysis of the crystalline. **(C)** Ions release profile of SiO_3_^2-^, Zn^2+^ or Sr^2+^. (*n* = 3).

The effect of sintered temperature on SZS scaffolds was firstly investigated. As shown in Figure 1A, the density of the ceramic increased gradually with the elevated sintered temperature from 1,200°C to 1,400°C. Although the XRD patterns demonstrated that the SZS phase (JCPDS card: no. 39–0,235) contributed to the main component of the scaffolds in all groups without apparent differences (Figure 1B), the concentration of the released ions (SiO_3_^2-^, Zn^2+^ or Sr^2+^) from the scaffolds was negatively correlated to sintering temperature (Figure 1C). In consideration of the possible cytotoxicity of excess ion release, SZS scaffolds sintered at 1,400°C were chosen for further studies.

### 3.2 Characterization of the 3D printed porous SZS scaffolds

**FIGURE 2:**
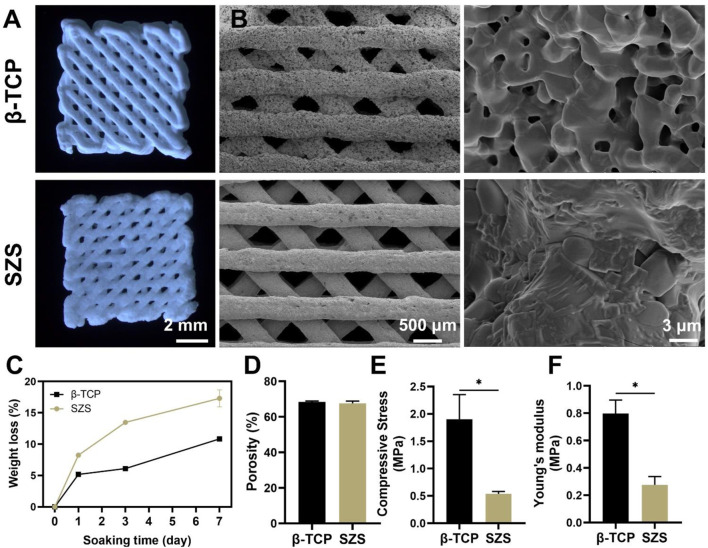
Characterization of 3D printed SZS and β-TCP scaffolds. **(A)** Optimal images. **(B)** SEM characterization of the macroporous structure and surface morphology. **(C)** *In vitro* degradation assay. (*n* = 3). **(D)** Porosity of scaffolds. (*n* = 4). **(E)** Compressive stress and **(F)** Young’s modulus of scaffolds. (*n* = 3). ^*^ *p* < 0.05 and ^**^ *p* < 0.01.

3D printed porous scaffolds of SZS and β-TCP with the uniform 45° interlaced architectures were shown by the optical images (Figure 2A). SEM images (Figure 2B) revealed that the pore size in both the scaffolds was about 450 μm. The porosities of the SZS and β-TCP scaffolds were 67.6 and 68.46%, respectively (Figure 2D). There are no significant differences in the macro pore structures between SZS and β-TCP scaffolds, indicating the excellent controllability of 3D printing technique. Furthermore, the mechanical properties of the scaffolds were evaluated, and the 3D printed β-TCP scaffolds showed higher compressive stress and Young’s modulus than the 3D printed SZS scaffolds (Figures 2E,F). Correspondingly, the *in vitro* degradation rate of SZS scaffolds was also faster than β-TCP scaffolds (Figure 2C), which reached 17.41% after 7 days.

### 3.3 Immunomodulation behavior of the 3D printed porous SZS scaffolds

**FIGURE 3:**
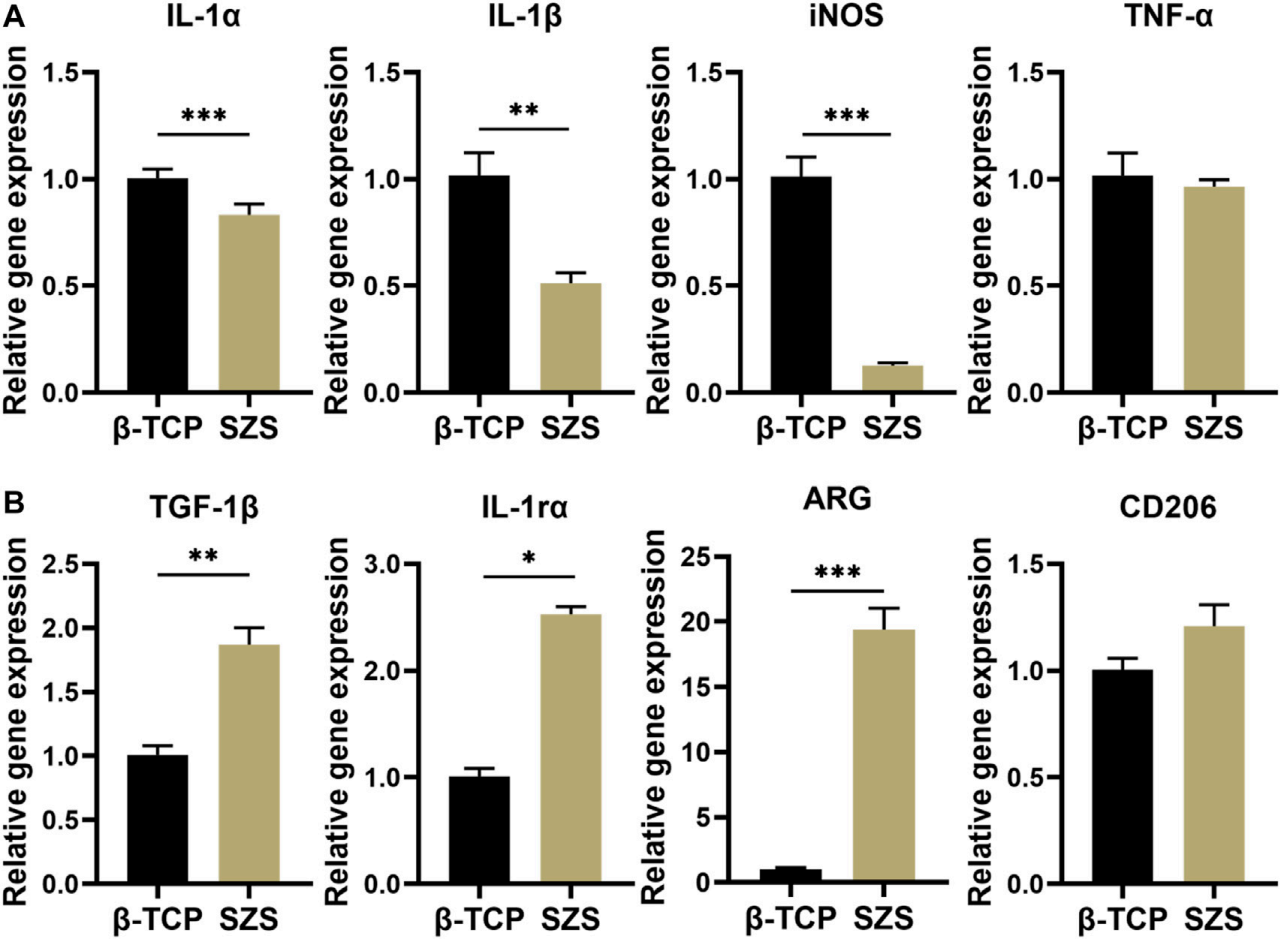
Gene expression of **(A)** inflammatory factors (IL-1α, IL-1β, iNOS, and TNF-α) (*n* = 4) and **(B)** pro-healing factors (TGF-1β, IL-1rα, ARG, and CD 206) in RAW264.7 macrophages after the treatment of different 3D printed scaffolds. (*n* = 4). ^*^ *p* < 0.05; ^**^ *p* < 0.01; ^***^ *p* < 0.001.

The cytotoxicity of scaffolds on RAW264.7 macrophages was first evaluated and the result was shown in Supplementary Figure S1. Both β-TCP and SZS scaffolds were non-toxic and SZS scaffolds could even promote the cell viability of macrophages. The mRNA expressions of inflammatory factors secreted from RAW264.7 macrophages in presence of the scaffolds were investigated and the results are shown in Figure 3. Downregulation of Pro-inflammatory factors such as IL-α, IL-1β and iNOS were exhibited in SZS group as compared to β-TCP group (Figure 3A). Also, significant upregulation of pro-healing factors after the treatment of SZS scaffolds was exhibited compared with β-TCP scaffolds, especially for factors of TGF-1β, IL-1rα, ARG (Figure 3B). All these results indicated that 3D printed SZS scaffolds had a beneficial pro-healing immune regulation ability.

### 3.4 Promotion of *in vitro* osteogenesis by the macrophage/scaffold conditional medium

**FIGURE 4:**
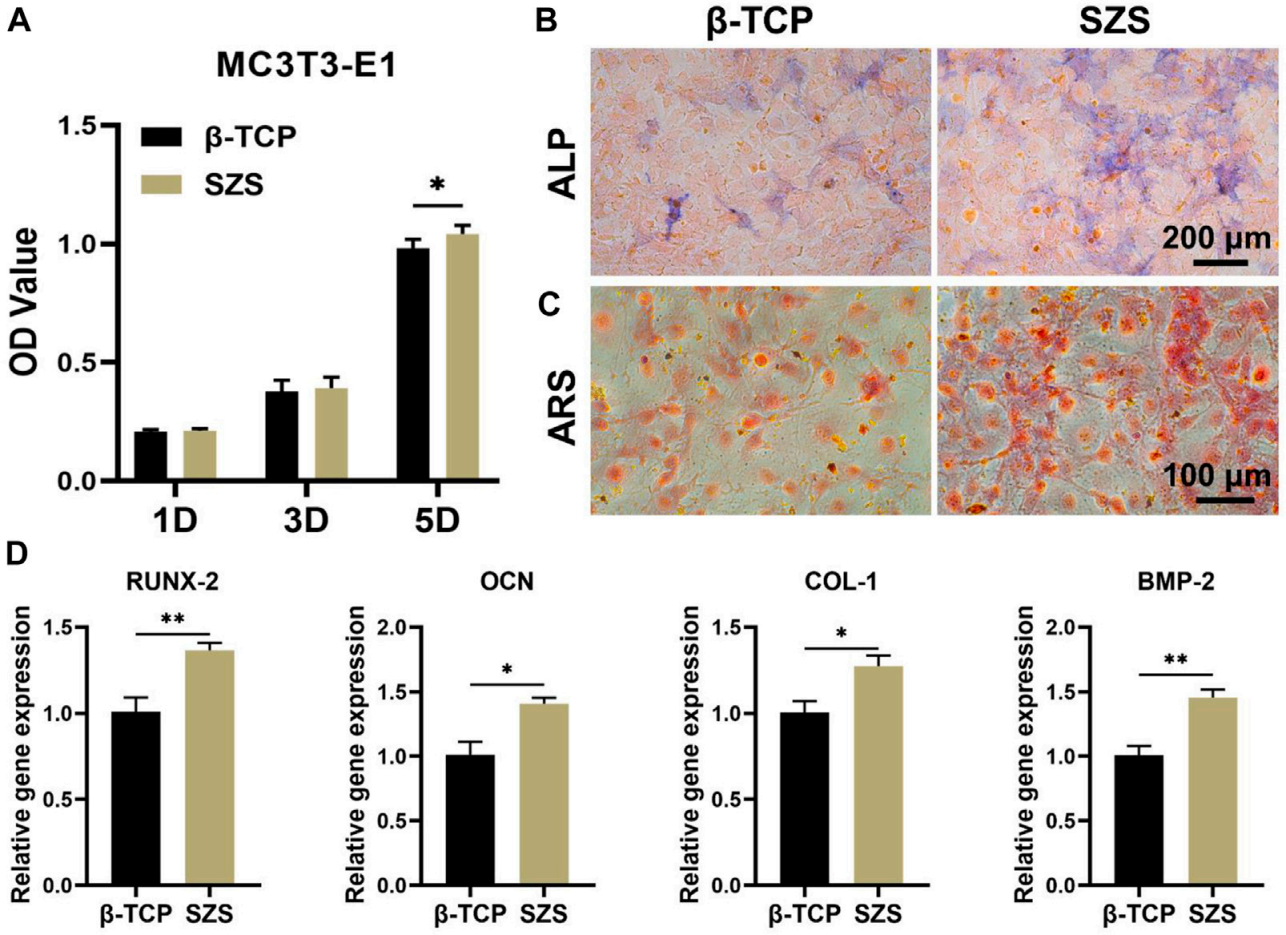
Effect of macrophage/scaffold conditional medium on MC3T3-E1 cells. **(A)** Cell proliferation on day 1, 3, and 5. (*n* = 6). **(B)** Representative images of ALP staining after 7 days’ culture. Blue color represents ALP. **(C)** Representative images of Alizarin red s (ARS) staining after 7 days’ culture. Orange-red color represents calcium nodules. **(D)** The osteogenic genes (RUNX-2, OCN, COL-1, and BMP-2) expression in cells by q-PCR assessment after 7 days’ culture. (*n* = 4). ^*^ *p* < 0.05; ^**^ *p* < 0.01.

To further evaluate the osteoimmunomodulation ability of the scaffolds, the macrophage/scaffold conditional medium was prepared. The cell proliferation results showed that more MC3T3-E1 cells proliferated after the culture with SZS mediated conditional medium as compared to β-TCP group, especially on day 5 since a significant difference was shown between SZS and β-TCP group (Figure 4A). Also, the ALP and alizarin red staining of cells treated with different conditional media for 7 days were conducted, which displayed that higher expression of ALP and more calcium nodules appeared in group SZS as compared to group β-TCP (Figures 4B,C). Moreover, the osteogenic gene expression of MC3T3-E1cells cultured with different conditional media for 7 days was estimated by the q-PCR method. As shown in Figure 4D, significantly higher expressions of COL-1, BMP-2, OCN, and RUNX-2 were observed in the SZS group than that in the β-TCP group. All these outcomes implied that SZS scaffolds had better osteoimmunomodulation activity than β-TCP scaffolds.

### 3.5 Promotion of *in vitro* angiogenesis by the macrophage/scaffold conditional medium

**FIGURE 5:**
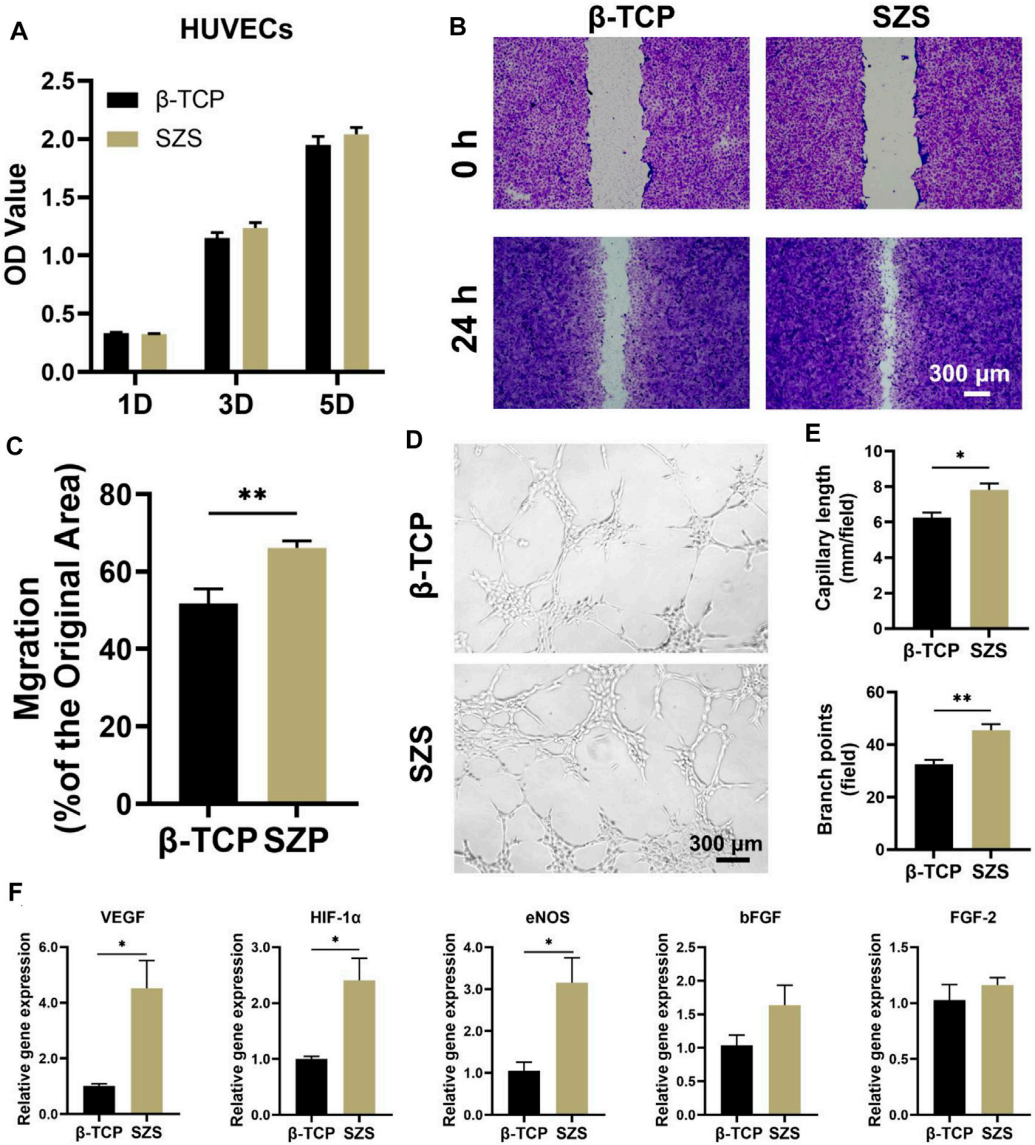
Effect of macrophage/scaffold conditional medium on HUVECs. **(A)** Cell proliferation on day 1, 3, and 5. (*n* = 6). **(B)** Representative images of cell migration after 1 day’s culture. **(C)** Quantitative analysis of cell migration rate. (*n* = 8 for β-TCP; *n* = 9 for SZS). **(D)** Representative images and **(E)** quantitative analysis of the *in vitro* tube formation. (*n* = 4). **(F)** The angiogenic genes (VEGF, HIF-1α, eNOS, bFGF and FGF-2) expression in cells by q-PCR assessment after 3 days’s culture. (*n* = 4). ^*^ *p* < 0.05; ^**^ *p* < 0.01.

Apart from the osteoimmunomodulation performance of the scaffolds, the immune-regulated angiogenic performance was also explored in our study. The effect of conditional medium on HUVECs was investigated including cell proliferation, migration, and differentiation. The cell proliferation study showed that a slight proliferative promotion of HUVECs was observed in the SZS group as compared to the β-TCP group without significant differences (Figure 5A). However, the cell migration rate in the SZS group was significantly higher than that in the SZS group (Figures 5B,C). More interestingly, the *in vitro* tube formation assay exhibited that the SZS group promoted more tubes formation in Matrigel after culture for 4 h as compared to the β-TCP group (Figure 5D). The corresponding quantitative analysis showed that the number of branch points and the capillary length in the SZS group was about 1.4 and 1.2 folds of that in group β-TCP, respectively (Figure 5E). Finally, significantly higher expression of angiogenic genes including VEGF, HIF-1α and eNOS in HUVECs were observed in the SZS group as compared to the β-TCP group (Figure 5F). The outcomes indicated that SZS scaffolds had a better immune regulation effect on the pro-angiogenesis of HUVECs than β-TCP scaffolds.

### 3.6 *In vivo* vascularized bone regeneration by the scaffolds in critical-sized calvarial defects

**FIGURE 6:**
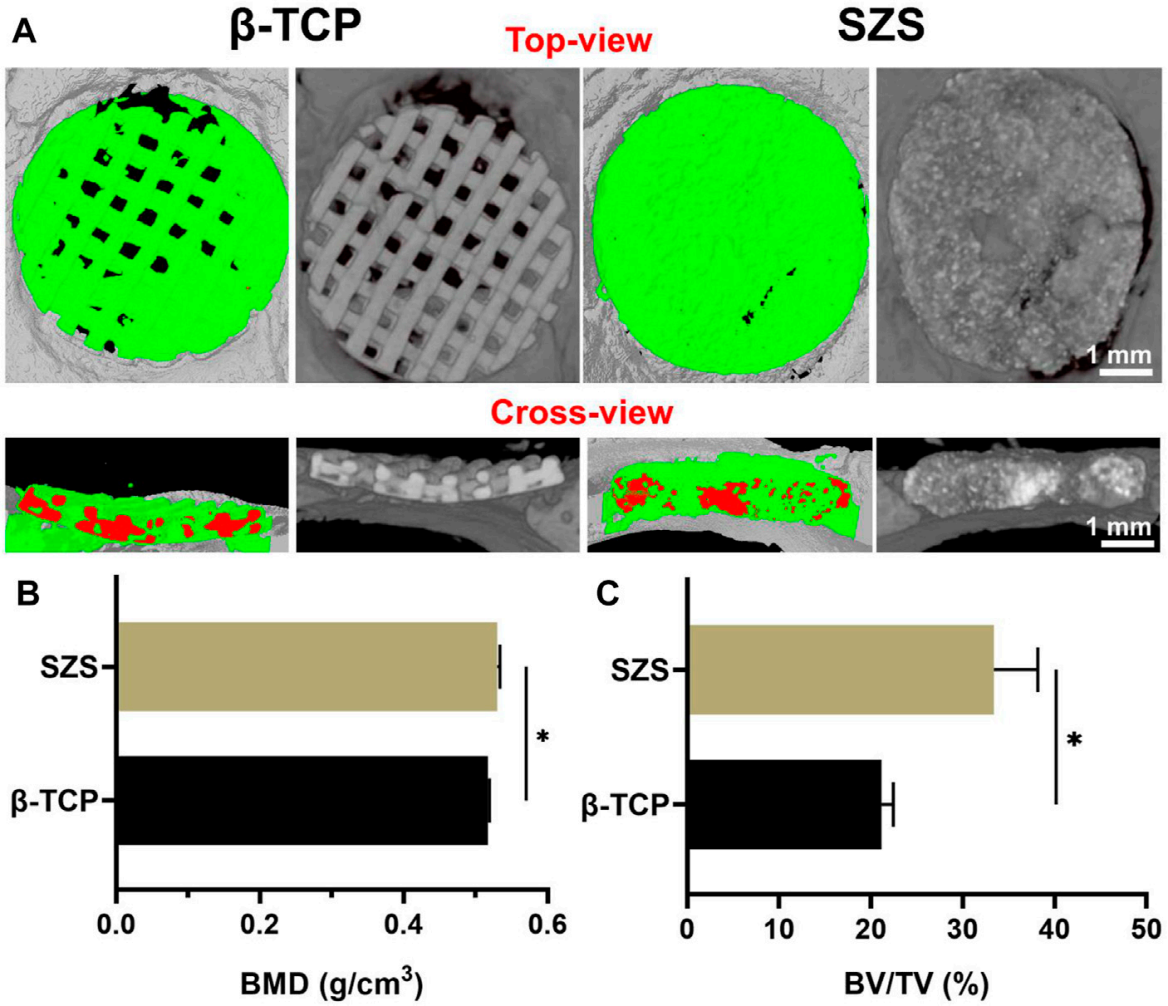
Micro-CT analysis of new bone formation in defect areas after implantation of 3D printed SZS and β-TCP scaffolds for 2 months **(A)** Typical 3D reconstruction of micro-CT images from the top view and cross-view. Green color shows newly formed bone, and red color represents materials. Quantitative analysis of **(B)** bone mineral density (BMD) (*n* = 4) and **(C)** bone volume/total volume (BV/TV) from micro-CT data. (*n* = 4). ^*^ *p* < 0.05.

**FIGURE 7:**
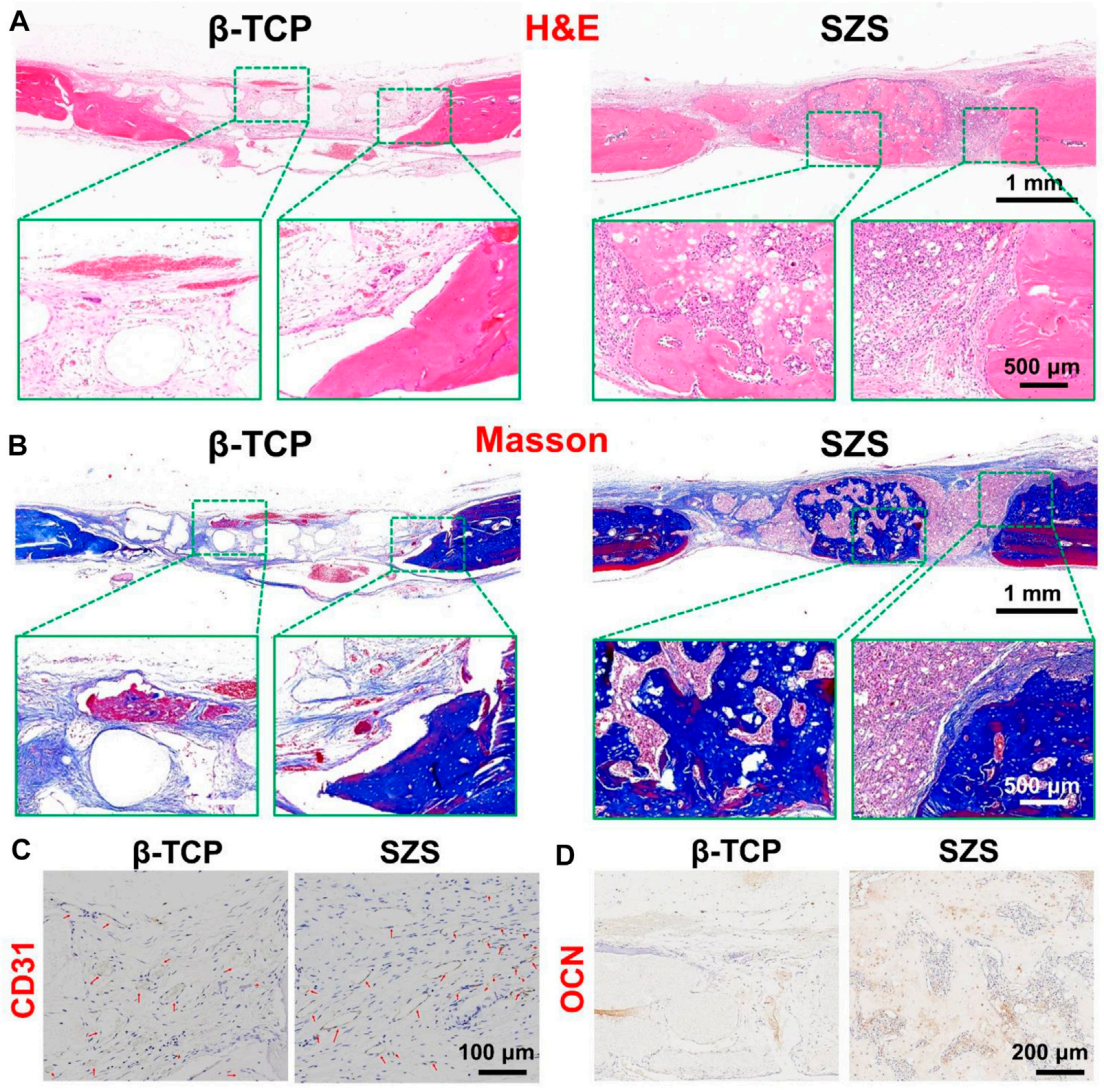
Representative H&E staining **(A)** and Masson’s trichrome staining **(B)** of the craniums with cranial defects after implantation of 3D printed SZS and β-TCP scaffolds for 2 months. Representative immunohistochemistry staining images targeting angiogenic marker CD31 **(C)** and osteogenic marker OCN **(D)** in new-formed tissues.

To further evaluated the *in vivo* bone regeneration ability of the 3D printed scaffolds, a typical rat calvarial defect model in rats was built and filled with both 3D printed β-TCP and β-TCP scaffolds for 2 months, respectively. The 3D reconstructed micro-CT images revealed that more new bone tissues were deposited in the SZS scaffolds compared with the β-TCP scaffolds, which was confirmed by the quantitative analysis of bone mineral density (BMD) and bone volume fraction (BV/TV) (Figures 6A–C). It is worth mentioning that SZS scaffolds degraded much faster than β-TCP scaffolds as the porous structure was fragmentized in the SZS group, whereas it was maintained in the β-TCP group. The histological analysis of H&E staining and Masson’s trichrome staining also showed consistent results where more newly formed tissues including collagen were found in defects treated with SZS scaffolds than those treated with β-TCP scaffolds (Figures 7A,B). Furthermore, the immunohistochemical staining of osteogenic biomarker OCN and angiogenic biomarker CD31 showed that SZS scaffolds had a better promotion of osteogenesis and angiogenesis in newly formed bone tissues (Figures 7C,D). All these results demonstrated that 3D printed SZS scaffolds had the bioactivity to promote vascularized bone regeneration.

## 4 Discussion

Scaffolds serve as a bridge guiding tissue regeneration in bone defects, which require essential mechanical properties and befitting porous structures. The technique of 3D printing emerged in the 1990s and has been proved as a critical fabrication method of bone scaffolds due to its flexibility in controlling the bulk geometry and internal structures, showing the ability in balancing mechanical properties and porous structures (Zhen et al., 2021). Here, both SZS and β-TCP scaffolds with high porosity were fabricated by 3D printing, which was demonstrated beneficial for bone regeneration (Indranath et al., 2021). Therefore, 3D printed SZS porous scaffolds might be available for bone regeneration. Apart from the structure, the component of scaffolds also affect the outcomes of bone repair. Silicate biomaterials usually show better bioactivity for the deposition of bone-like apatite compared with phosphate biomaterials such as HA and β-TCP due to the exposed siloxane groups, and the commercial products of silicate bioglass (e.g., 45S5Bioglass^®^ and BonAlive^®^) have been widely used in the field of bone regeneration for many years (Cannio et al., 2021). Recently, increasing evidences show that the released bioactive ions including SiO_3_^2-^ ions and the corresponding metal ions (e.g., Ca^2+^, Mg^2+,^ and Sr^2+^) from the silicate biomaterials make the main contributions to biological activities such as osteogenesis and angiogenesis (Yang et al., 2021a; Dong et al., 2022; Fan et al., 2022). It is revealed that SiO_3_^2-^ ions could stimulate osteogenesis through multiple signaling pathways including Wnt/β-catenin, Shh, MAPK/Erk, and antagonizing nuclear factor kappa-B (NF-κB) signaling pathways (Han et al., 2013; Shie and Ding, 2013; Guan et al., 2015; Mao et al., 2017). Also, SiO_3_^2-^ ions could stimulate angiogenesis by upregulating the expression of pro-angiogenic factors such as VEGF, bFGF and HIF-1α (Li and Chang, 2013; Xu et al., 2021; Que et al., 2022). More interestingly, the combination of SiO_3_^2-^ ions with other bioactive ions may result in a synergetic effect on both osteogenesis and angiogenesis (Mao et al., 2017; Xing et al., 2018).

However, most studies focused on the direct effects of silicate biomaterials on bone relative cells and ignored the relationship between the immune system and the skeleton system. As a matter of fact, immune cells first respond to the implanted biomaterials among all types of cells and play an indispensable role in the following tissue healing process by collaboration with other cells. Taking the most common studied immune cells such as macrophages as an example, they are aggregated onto the surface of the materials when scaffolds are implanted, stimulated by the local microenvironment, and then release specific chemokines or cytokines to affect the behaviors of other cells such as osteoblasts and endothelial cells involved in bone regeneration (Chen et al., 2016). Our study proved that 3D printed SZS scaffolds could significantly increase the anti-inflammatory cytokines such as TGF-1β but decrease the pro-inflammatory cytokines such as IL-1α secreted from macrophages, showing a simulation of a pro-healing immune microenvironment. The following studies using the conditioned medium to culture MC3T3-E1 cells and HUVECs confirmed the beneficial immunomodulation effects on osteogenesis and angiogenesis with the treatment of 3D printed SZS scaffolds as compared to β-TCP scaffolds. The mechanism might be ascribed to the sustained release of the bioactive ions (SiO_3_^2-^, Zn^2+^ and Sr^2+^) from SZS as either SiO_3_^2-^, Zn^2+^ or Sr^2+^ exhibits a regulatory effect on immune cells depending on the concentrations (Chen et al., 2014; Zhong et al., 2022). Since the ions released in our study were in the active concentration range, their combination may result in higher efficiency in stimulating the pro-healing immune microenvironment for bone regeneration, which was also proved by the *in vivo* study as more new vascularized bone formed in 3D printed SZS scaffolds as compared to β-TCP scaffold. Our study demonstrated that 3D printed bioceramic scaffolds containing suitable nutrient elements could regulate the inflammatory response of macrophages and further prompt osteogenesis and angiogenesis. However, the disadvantages of SZS scaffolds are also apparent such as the relatively low compressive mechanical strength and fast degradation rate, which should be seriously considered in future applications.

## 5 Conclusion

In summary, porous SZS scaffolds were successfully fabricated using the 3D printing technique. The obtained SZS scaffolds could regulate the inflammatory response of macrophages and create a beneficial immune microenvironment for bone regeneration. Thus, the osteogenic activity of MC3T3-E1 cells and the angiogenic activity of HUVECs could be enhanced by the scaffold/macrophage conditioned medium. In addition, the *in vivo* study of skull defect model in rats demonstrated the excellent vascularized bone reconstruction performance of 3D printed SZS scaffolds. Our results suggested that 3D printed porous SZS scaffolds have the potential for repairing large bone defects.

## Data Availability

The original contributions presented in the study are included in the article/Supplementary Material, further inquiries can be directed to the corresponding authors.
