# Supplementary material for: 3D-printed Sr2ZnSi2O7 scaffold facilitates vascularized bone regeneration through macrophage immunomodulation
Source: Front Bioeng Biotechnol. 2022 Sep 16;10:1007535. doi: 10.3389/fbioe.2022.1007535 (PMC9523139; doi:10.3389/fbioe.2022.1007535)
Supplement: Supplementary file 1 [file DataSheet1.docx]

3D-printed Sr_2_ZnSi_2_O_7_ scaffold facilitates vascularized bone regeneration through macrophage immunomodulation

**Supplemental Figure:**


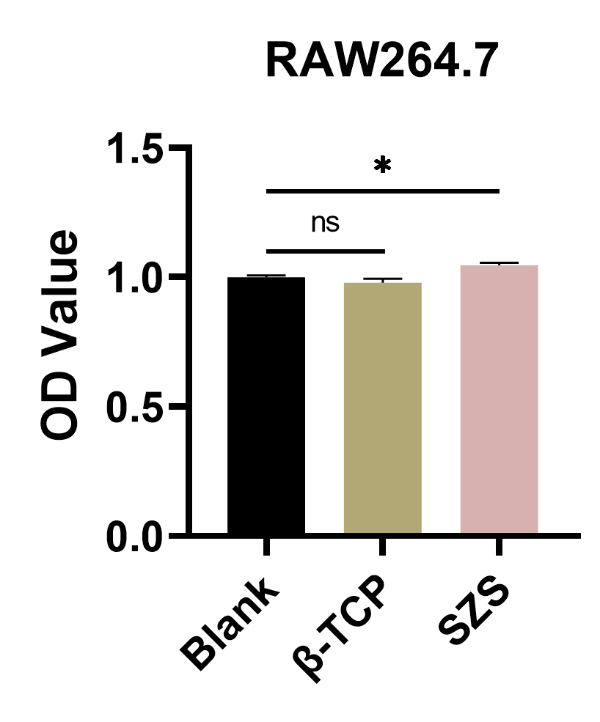


**Figure S1:** The cytotoxicity of scaffolds on RAW264.7 macrophages. (n=4). ns: no significant difference; *P < 0.05.
